# Effects of Tai Chi on Self-Efficacy: A Systematic Review

**DOI:** 10.1155/2018/1701372

**Published:** 2018-08-15

**Authors:** Yingge Tong, Ling Chai, Song Lei, Miaomiao Liu, Lei Yang

**Affiliations:** ^1^Medical School, Hangzhou Normal University, Hangzhou, Zhejiang 310036, China; ^2^Medical School, Shihezi University, Shihezi, Xinjiang 832003, China

## Abstract

The purpose of this systematic review is to summarize and update the readers regarding clinical studies that have investigated the effects of Tai Chi on self-efficacy and to describe their limitations and biases. Nine electronic databases were searched from the establishment of the database until August 10, 2017. All randomized controlled trials (RCTs), nonrandomized controlled studies (NRSs), quasi-experimental studies, or studies with pre-post design were included if they clearly defined a Tai Chi intervention and evaluated self-efficacy outcomes. We categorized these 27 studies into the “disease category” and the “population category,” based on the types of participants. This systematic review summarizes the effects of Tai Chi on self-efficacy in various populations and found that Tai Chi appeared to have positive effects on self-efficacy in some populations. Fifteen research studies showed that Tai Chi had significant positive effects on self-efficacy, while 11 studies did not; only one study found a negative outcome at the follow-up. In addition, it is unclear which type, frequency, and duration of Tai Chi intervention most effectively enhanced self-efficacy. Tai Chi appears to be associated with improvements in self-efficacy. Definitive conclusions were limited due to the variation in study designs, type of Tai Chi intervention, and frequency, and further high-quality studies are required.

## 1. Introduction

Tai Chi, an ancient Chinese healing/martial art, combines martial art movement with Qi (vital energy circulation), breathing, and stretching techniques. It has evolved into five different styles over the course of its development: Chen, Yang, Sun, Wu, and W'u [1]. It offers physiological and psychological benefits and improves quality of life in various populations. A growing body of evidence suggests that Tai Chi improves balance, aerobic capacity, muscular strength, and flexibility and can relieve psychological problems such as depression, anxiety, stress, and mood disturbance [2–6]. Studies have provided evidence that psychological variables, such as self-efficacy, predict behavioral adherence. Self-efficacy is positioned early in a causal chain of factors that are directed to determine behavior [7]. Many studies have explored the influence of self-efficacy on behaviors and physical functioning, such as smoking cessation, physical activity, healthy eating, and weight management [8–10]. High level of self-efficacy has positive effects on mental health and quality of life. Self-efficacy is a psychological construct based on social cognitive theory and it describes the interaction between behavioral, personal, and environmental factors in health and chronic disease. The theory of self-efficacy proposes that the confidence in one's capability to perform certain health behaviors influences a patient's engagement in and performance of those behaviors, which in turn influences health outcomes. The concept of self-efficacy can be domain- or behavior-specific (specific self-efficacy) or may involve global beliefs surrounding self-efficacy without specific conditions (general self-efficacy) [11–13]. Importantly, self-efficacy is not a static trait but a modifiable characteristic. It can be altered, and enhanced self-efficacy can be associated with improved health status in the areas affected by those specific behaviors [14].

Some studies have provided evidence that Tai Chi interventions can improve the self-efficacy of patients with specific diseases, such as knee osteoarthritis (KOA) [15], Chronic Obstructive Pulmonary Disease (COPD) [16], and chronic heart failure [17]. Some studies also reported that Tai Chi had positive effects of self-efficacy in certain populations, such as college students [18] and elderly people [19]. One review [20] showed that Tai Chi could enhance overall psychological well-being and improve self-efficacy, but it was conducted in 2007 and used “mental health,” “mood,” “depression,” “self-efficacy,” and other keywords that emphasized the mental/psychological health effects of Tai Chi practice in different populations. Despite the popularity of Tai Chi, the overall effects of Tai Chi intervention on self-efficacy are unclear or have not been recently updated. We conducted this study to systematically review the effects of Tai Chi on domain-specific and general self-efficacy in different populations and to identify the limitations and biases of these published clinical research studies. The findings of this review may find applications in rehabilitation initiatives and provide new directions for research.

## 2. Methods and Materials

### 2.1. Data Sources and Searching Strategies

The aims and methods of this systematic review were registered with the PROSPERO database prior to conducting the review (#CRD 42017078861). A study protocol accompanied by a data extraction form was formulated and critically reviewed by two experts prior to the initiation of this study. Relevant studies were searched and retrieved from nine electronic databases, which included five English databases: PubMed, Web of Science, EMBASE, EBSCO, Cochrane Database and four major Chinese databases: China National Knowledge Infrastructure (CNKI), Wan Fang Data, Chinese Scientific Journal Database (VIP), and Sino Med. Two reviewers (Ling Chai, Yingge Tong) independently searched the literature according to the study protocol. The key words used included combination of MESH and free text terms, such as “Tai Chi,” “Tai Ji,” “Personality,” “Self Efficacy,” “Self Esteem,” “Self Perception,” and “Self Concept,” were used in the search strategies.

### 2.2. Inclusion and Exclusion Criteria

Inclusion and exclusion criteria for this systematic review included types of studies, participants and interventions, primary outcomes, and language.* (1) Types of Studies.* Eligible study designs included randomized controlled trials (RCTs), nonrandomized controlled studies (NRSs), quasi-experimental studies, and studies with pre-post design. We excluded studies which used qualitative methods, reviews, case studies, and conference proceedings that did not provide primary data.* (2) Types of Participants*. We included subjects who were physically capable of practicing Tai Chi, whether they were healthy or ill at baseline; there were no specific restrictions on the participants' age, gender, race, or health status.* (3) Types of Interventions*. The interventional measures were a single Tai Chi intervention or Tai Chi integrated with baseline interventions (e.g., health education or usual care) which were equally implemented in the control group. Trials compared Tai Chi with or without baseline interventions to usual physical activity, waiting-list control, or blank control.* (4) Primary Outcomes*. The concept of self-efficacy in our research included general self-efficacy and domain-specific self-efficacy.* (5) Language Restrictions*. English and Chinese language papers were considered.

### 2.3. Study Selection and Data Extraction

We independently performed the initial selection for eligibility based on titles and abstracts related to Tai Chi exercise and the psychological/mental variables of self-efficacy. Of the 824 records, we screened 541 abstracts after excluding duplicates. We excluded 496 records; among these, 427 were irrelevant, 58 were reviews, and another 11 articles included surveys, commentary, case studies, and conferences proceedings. When data were not provided in publications, we contacted the authors for information. Two reviewers extracted data and assessed the trial quality of each study independently. Disagreements were resolved by consensus.

Study characteristics and outcome data of each included trial were extracted with our data extraction form, which included (1) first author, year of publication, country, study design, and setting, (2) participant characteristics (age, sample size), (3) intervention protocols (types of Tai Chi, intervention frequency, and duration of Tai Chi exercise) and types of controls, and (4) outcome measures, main results, and conclusion.

### 2.4. Quality Assessment for the Included Studies

Three authors independently evaluated the methodological quality of the RCTs based on the Jadad instrument, which is easy to use and describes randomization, blinding, and withdrawals/dropouts. Studies were graded by adding points for the above-mentioned items. The highest score was five, indicating the highest-quality study [21, 22].

## 3. Results

### 3.1. Study Description

**Figure 1:**
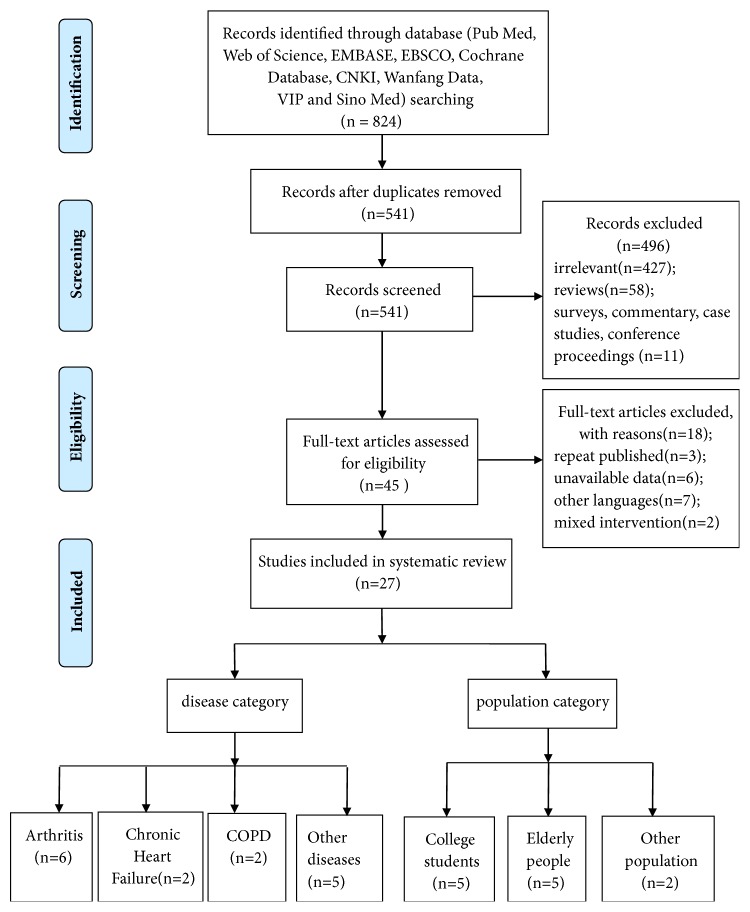
Flow diagram of study selection and identification.

We reviewed 541 English and Chinese articles and excluded 496 as they were irrelevant (n=427), were literature reviews (n=58), or were another type of publication such as a survey, case study, conference proceeding, or commentary (n=11). Finally, we retrieved 45 full-text articles for detailed evaluation, and 18 studies were eliminated as they were repeat publications (n=3), were in other languages (n=7), or had unavailable data (n=6) or their intervention did not meet the inclusion criteria for a systematic review (n=2). Therefore, 27 studies in total were identified for analysis. Three studies were identified from Chinese databases and 24 were from English databases (Figure 1).

### 3.2. Study Characteristics

The basic characteristics of the included studies were summarized in Table 1. This research included 20 RCTs. All the studies reported on randomization; 15 appropriately described the randomization method, while the remaining five did not report the method of randomization. Ten reported on blinding, and outcome assessors were blinded; the other ten did not mention whether the experimenters were blinded. Out of the 20 RCTs, 15 studies described withdrawals and dropouts. In addition, this systematic review included three NRSs, three quasi-experimental studies, and one pre-post clinical trial.

Fifteen studies used the Yang style; three and two studies adopted the Sun style and the Chen style, respectively; one study examined seated simplified Tai Chi; one study conducted wheelchair Tai Chi; and the remaining five did not clearly describe the type of Tai Chi. The length of each Tai Chi session varied in different studies, lasting from 15 to 120 minutes; the frequency of intervention was one to five sessions weekly. The most commonly used schedule was 60-minute sessions twice a week, which occurred in 11 studies. The duration of the intervention ranged from six to 26 weeks. The most common duration was twelve weeks, which was adopted by 16 studies (Table 1). In most studies, Tai Chi was taught by certified and experienced instructors or teachers (n=22); it was occasionally taught by researchers (n=1) or physiotherapists (n=1), and the remaining studies did not mention the instructor (n=3).

### 3.3. Effects of Tai Chi

Twenty-seven articles were organized into “disease category” and “population category,” according to the characteristics of the participants.

#### 3.3.1. Effects of Tai Chi on Different Diseases

There were 15 articles in the “disease category.” Six studies investigated patients with arthritis, two studies focused on patients with COPD, two studies investigated patients with chronic heart failure, and the remaining five studied patients with obesity, fibromyalgia, Parkinson's disease, and other diseases (Table 1).

*(1) Effects of Tai Chi on Arthritis*. We reviewed six studies examining the effects of Tai Chi on arthritis [15, 23–27], which included five RCTs and one pre-post clinical trial [26] and comprised a total of 732 subjects.

One study adopted Yang-style Tai Chi [24] while another three studies adopted 24-form simplified Yang style [26], 9-form Yang style [23], or 10-form classic Yang style [15]. The other two studies adopted Sun style [27] and 12-form Sun-style Tai Chi [25]. The duration of the Tai Chi intervention ranged from six to 12 weeks, with 60 minutes per session one to three times weekly. The control methods of the five studies included physical therapy, usual activities, education and stretching program, a wait-listed control (who were on a wait-list during the study and received an intervention after the study period had ended), and a true control group, which did not receive any treatment at all.

There were various measurements of self-efficacy in these six studies. Three studies used the Arthritis Self-Efficacy Scale (ASES) [14], which consisted of three domains: pain, function, and other symptoms. One study used the CPSS [28], which measures self-efficacy for pain management, physical function, and coping with symptoms. One study used the Motivation Scale for Health Behaviors [29], which comprised the variables of perceived self-efficacy, and one study used the 1-5 Self-Efficacy Scale [30].

The results of these six studies were varied. Two RCTs indicated that Tai Chi had significant positive effects on self-efficacy compared with the controls. The first one was conducted in the USA and included 40 symptomatic knee osteoarthritis participants, and it concluded that Tai Chi improved self-efficacy at 12, 24, and 48 weeks of follow-up [15]. The second study included 33 osteoarthritis patients, who showed improvements in self-efficacy for arthritis symptoms and total arthritis self-efficacy [23].

However, another RCT showed different results. It randomized 343 community participants to an eight-week Tai Chi intervention group or a wait-list control group. The arthritis self-efficacy for pain and other symptom management did not change significantly at the eight-week intervention and decreased at one year's follow-up [27].

Three studies failed to find significant improvement in self-efficacy. One RCT randomized 204 participants into the Tai Chi group or a standard physical therapy group [24]. And another RCT conducted in Korea in 72 osteoarthritis patients. Both studies showed that while self-efficacy improved in the Tai Chi group, this was not statistically significant compared with the controls [25]. The third study was a pre-post clinical trial, and no change was observed in the self-efficacy of pain management, physical function, and other symptoms after Tai Chi intervention [26].

*(2) Effects of Tai Chi on Chronic Heart Failure*. The same research team conducted two RCT studies [17, 31] in the United States to evaluate the effects of Tai Chi on self-efficacy in chronic heart failure patients. Both studies adopted Master Cheng Man-Ching's Yang-style short form, and the intervention comprised 12 weeks of 60-minute sessions twice a week. These studies used the Cardiac Exercise Self-Efficacy Instrument [17] and the Self-Efficacy-Barriers to Exercise Scale [31] to measure exercise self-efficacy. One RCT evaluated 100 subjects who had chronic systolic heart failure [17]. The other RCT evaluated 16 participants who had heart failure with preserved ejection fraction [31]. One RCT provided education to the participants in the control group [17], while, in the other study, those in the control group performed aerobic exercise [31]. One RCT showed that Tai Chi improved exercise self-efficacy significantly compared with the control [17], while the other concluded that self-efficacy improved after Tai Chi intervention but did not differ significantly between the Tai Chi group and the aerobic exercise group [31].

*(3) Effects of Tai Chi on COPD*. Two RCTs were conducted in the United States and Hong Kong that evaluated the effects of Tai Chi on self-efficacy in patients with COPD [16, 32]. The research carried out in the United States adopted Master Cheng Man-Ching's Yang-style short form, with 60 minutes per session twice a week for 12 weeks. The measurements were performed with the COPD-CSES and the SEMSOB [16]. The other study adopted Sun-style Tai Chi, with 15 minutes per session twice a week for six weeks, and reported results with the COPD-CSES [32]. The study in the USA divided 10 patients with moderate to severe COPD into the intervention group (Tai Chi plus usual care, n=5) and the control group (usual care, n=5). The results indicated that COPD self-efficacy improved in Tai Chi group but no significant difference between the two groups [32]. The other study recruited 192 COPD patients who consented to randomization to either a pulmonary rehabilitation program group (PRP) or a group with Tai Chi elements added to the PRP. The COPD self-efficacy and the SEMSOB of both groups improved significantly at the 6-month follow-up, but no comparison result between the two groups [16].

*(4) Effects of Tai Chi on Other Diseases*. Five studies evaluated the effects of Tai Chi on other diseases which were not included above. The first study was a RCT that focused on sedentary obese women who were allocated to either a 2-hour weekly group session of Tai Chi or a conventional structured exercise program, both of which lasted for 10 weeks. The results indicated that general self-efficacy as measured by the GSE Scale was improved in both groups and was maintained at the 30-week follow-up, but there is no significant difference between two groups [33].

The second RCT focused on fibromyalgia patients. The experimental group participated in a 12-week 8-form Yang-style Tai Chi program which was organized into 90-minute sessions twice weekly, while the control group received education. The results showed that scores on the three subsets of the ASES: arthritis pain self-efficacy, self-efficacy of physical function, and other symptoms all improved significantly in the experimental group compared with the control [34].

The third study was a RCT which focused on 60 patients with Parkinson's disease [35]. Routine neurological medical care was given to the control group, while the intervention group received an additional 12 weeks of Tai Chi. Scores on the MFES were significantly improved after Tai Chi intervention compared with the control group.

Two studies focused on elderly people with other diseases. One NRS studied frail, institutionalized elderly people who either were healthy or had arthritis, DM, hypertension, or other complicated diseases. The participants took part in either a 24-week Tai Chi intervention implemented four days weekly or a cognition-action (CA) exercise program implemented for 30 minutes twice weekly. Scores on the Falls Efficacy Scale and TCSE scale were enhanced significantly in both groups, but no significant difference between two groups [36]. The other was a quasi-experimental pre-post study that recruited elderly Korean American women with Alzheimer's disease, other forms of dementia, depression, bipolar disorder, and other diseases. The control group received health education, while the intervention group received health education plus a weekly Tai Chi intervention for 60 minutes weekly over 16 weeks. The result indicated self-efficacy increased after intervention, no significant difference between the two groups [37].

#### 3.3.2. Effects of Tai Chi on Different Populations

There were 12 articles under the “population category.” Five focused on college students, while another five studies investigated the elderly population. Four studies focused on inactive and disabled elderly people, and one study investigated healthy elderly people. The remaining two studies investigated healthy adult participants and ethnic Chinese adults with cardiovascular disease (CVD) risk factors.

*(1) Effects of Tai Chi on College Students*. Five studies, including three RCTs [38–40], one NRS [18], and one quasi-experimental study [41], focused on the effect of Tai Chi intervention in college students. Overall, 799 college students were recruited, with 339 allocated into intervention groups and 460 into control groups.

Two studies adopted 24-form simplified Tai Chi [38, 39], another two adopted Chen-style Tai Chi [18, 41], and the remaining study did not describe the type of Tai Chi intervention that was performed [40]. The interventions lasted from 12 to 15 weeks, with 50 to 60 minutes per session two to five times weekly.

In these studies, two different concepts of self-efficacy were measured with different scales. Three studies measured the concept of general self-efficacy with the GSE Scale [39, 40] and the Chinese adaptation of the GSE Scales [38]. Another two used the Self-Regulatory Self-Efficacy Scale [18, 41] to measure exercise self-efficacy.

Among the five studies, three revealed that Tai Chi did not significantly improve self-efficacy. One RCT allocated 198 college students into the Tai Chi group and the usual physical activities group, and no significant changes were found after 12-week intervention and the comparison of two groups [38]. Another RCT recruited 206 college students, and although GSE Scale scores improved in the Tai Chi group, there was no significant difference between the experimental and control groups [39]. A quasi-experimental study compared the effect of 15-week Tai Chi courses (experimental group, n=76) and special recreation (control group, n=132), showing no difference between the two groups [41].

On the other hand, two researchers found that self-efficacy significantly improved after Tai Chi intervention. A NRS recruited 127 college students from the United States, showing that self-efficacy significantly improved after attending 15 weeks of Tai Chi classes [18]. Similarly, a RCT recruited 60 Chinese college students and showed that self-efficacy significantly improved in the experimental group after a 12-week intervention, as compared to the group that did other activities [40].

*(2) Effects of Tai Chi on the Elderly*. One RCT [42] and one NRS [43] assessed the effects of Tai Chi on self-efficacy in disabled elderly people in wheelchairs or with a disability, respectively. Two other RCTs studied inactive elderly adults [19, 44], while an NRS studied healthy elderly subjects [45]. In the first three studies [19, 44, 45], 24-Form Yang-style Tai Chi was adopted, whereas seated simplified Tai Chi [42] and Wheelchair Tai Chi [43] were adopted once each in the other two studies. The duration of Tai Chi intervention ranged from six to 26 weeks, with 40 to 70 minutes per session one to three times per week.

In these studies, three different concepts of self-efficacy were measured with different scales. Fall self-efficacy was measured with ABC scale [46] and the Falls Self-Efficacy Scale [47]. Exercise self-efficacy was measured with Self-Efficacy for Exercise (SEE) scale [48] and the Exercise-related Self-Efficacy Scale [19], and pain self-efficacy was measured with PSEQ [49].

Two studies, including one RCT and one NRS, focused on disabled people [42, 43]. The RCT compared the effects of seated Tai Chi exercise and usual standard activities on the self-efficacy of older people living in a long-term care facility and using wheelchairs for mobilization. After 26 weeks of intervention, the Tai Chi group recorded significantly higher exercise self-efficacy levels than the control group [42]. The NRS recruited 40 disabled elderly people, conducting a 12-week Wheelchair Tai Chi 10-Form intervention; it indicated that pain self-efficacy significantly improved after Tai Chi intervention, with no comparisons between the two groups [43].

Two studies investigated elderly people with low activity levels and indicated that Tai Chi had significant positive effects on self-efficacy compared with the controls. One RCT involved 256 community-dwelling older adults who participated in either a Tai Chi group (n=125) or a stretching control exercise group (n=131) for twenty-six weeks, showing that Tai Chi improved significantly falls self-efficacy [44]. In the other RCT [19], healthy subjects with low activity levels were randomly assigned to either a Tai Chi group or a wait-list control group for six months, and Tai Chi significantly improved barriers efficacy and performance efficacy.

A NRS [45] investigated the potential value of Tai Chi in improving falls self-efficacy in a sample of healthy elderly people, and it was observed that Tai Chi did not have a significant positive effect on falls self-efficacy scores.

*(3) Effects of Tai Chi on Healthy Adults and a CVD High Risk Group*. One RCT [50] evaluated the effects of Tai Chi on the self-efficacy of 70 healthy adults in Switzerland, in which the participants were randomly allocated to the intervention group or the waiting-list control group for 12 weeks. The Tai Chi group showed a higher increase in general self-efficacy as measured by the GSE Scale after intervention.

In a quasi-experimental study [51] with a 12-week Tai Chi exercise intervention in ethnic Chinese people with CVD risk factors living in the United States, participants attended 60-minute Tai Chi sessions three times per week for 12 weeks. The results revealed that Tai Chi significantly increased the self-efficacy of overcoming barriers to Tai Chi and confidence in performing Tai Chi, which were measured by the TCSE scale.

## 4. Discussion

We conducted a systematic review to summarize and update the readers on studies that have investigated the effects Tai Chi on self-efficacy and to describe the limitations and biases of these clinical studies.

### 4.1. Summary of Review Results

Tai Chi, an important feature of traditional Chinese art, has spread worldwide over the past two decades. Self-efficacy is a determinant of life outcomes both directly and by its influence on other psychological, social, and behavioral factors [52]. This systematic review summarizes the effects of Tai Chi on self-efficacy in various populations and it suggests that Tai Chi may have positive effects on self-efficacy of some populations. We included both English and Chinese studies, as Tai Chi originated in China and many studies are published in the Chinese language. We searched five English and four Chinese databases, retrieved 824 studies, and finally enrolled 27 related studies. Although “self-efficacy” has impact on “mental health” and “quality of life,” the meaning of the three concepts are different. In the enrolled 27 studies, three studies [15, 19, 23] showed that enhanced self-efficacy, together with other variables, such as increased social support and reduced tension, may have mediated increased satisfaction with general health and led to improved physical, psychological, and psychosocial well-being and overall quality of life.

Among the 27 studies, 15 showed a significant increase in self-efficacy through Tai Chi intervention (Table 2). Among the 15 studies, 10 showed significantly increased self-efficacy of the Tai Chi groups compared with the control groups. Four studies showed significantly improved self-efficacy of Tai Chi groups after intervention; however, the between-group comparisons were inconsistent: two of them showed no significant differences between Tai Chi groups and the control groups, and another two did not mention the comparison result of the two groups. Among the 15 studies, one was a pre-post comparison study.

Social cognitive theory is based on an interactional model of human behavior, helpful for understanding the dynamic nature of health behavior. According to the theory, individuals rely on four sources of information to improve self-efficacy, which are mastery experiences, vicarious learning, verbal or social persuasion, and physiological and affective status [51, 53]. Among the 15 studies, two studies [18, 51] mentioned that Tai Chi enhanced self-efficacy through mastery experiences, vicarious learning, verbal or social persuasion, and physiological and affective status. Two other studies [19, 23] showed that Tai Chi provided a mastery experience that increased participants' confidence about their ability to manage their symptoms.

Other findings provide preliminary insight into one mechanism of how Tai Chi may contribute to health promotion. The mediating role of general self-efficacy in Tai Chi-induced reduction in perceived stress was explored in a previous study [50]. Another study showed that self-efficacy regarding falls mediates the fear of falling in Tai Chi intervention [44]. In the present systematic review, two studies [35, 44] reflected this finding. One study explored the mediating role of general self-efficacy in Tai Chi -induced reduction in perceived stress [50].

Among the 27 studies, 11 studies showed no significant differences between the intervention and control groups, or Tai Chi groups showed no changes after intervention (Table 3). Among the 11 studies, seven showed that self-efficacy improved after Tai Chi intervention but found no significant differences compared with the control groups; three studies found that Tai Chi groups showed no changes after intervention and no significant differences between Tai Chi groups and the control groups. One pre-post study showed that no change was found in Tai Chi group after intervention.

Among the 27 studies, only one study showed that the self-efficacy of the Tai Chi group did not show significant changes after 8 weeks of intervention and the effect decreased at one-year follow-up (Table 3).

There were several possible explanations for these results. First, the intervention group had higher self-efficacy levels than the control group did before the intervention. Second, the samples were small, with 13-35 in Tai Chi groups and 5-34 in the control groups. Third, the measurement of the study may not be appropriate for the population. Fourth, Tai Chi intervention was not long enough to produce significant changes in self-efficacy. Fifth, the participants enrolled were young and apparently healthy people, which may result in nonobvious results.

To more specifically analyze and compare these studies, we divided the 27 articles into the “disease category” and “population category,” comparing the effect of Tai Chi on self-efficacy in different populations according to the characteristics of the participants.

### 4.2. Effects of Tai Chi on the Self-Efficacy of the Participants with Different Diseases

Under the “disease category” 15 studies analyzed the effects of Tai Chi on the self-efficacy of patients with arthritis, chronic heart failure, COPD, and other diseases, but they did not show consistent results.

(1) Six studies analyzed the effects of Tai Chi on the self-efficacy of arthritis patients [15, 23–27]. Two RCTs with Jadad scale scores of three [23] and four [15] indicated that Tai Chi groups had significant improvement on self-efficacy compared with the control groups.

Among the six studies, another two RCTs with quality scores of four [24] and three [25] measured by the Jadad scale showed that the Tai Chi groups had improvement on self-efficacy, but no significant differences compared with the control groups. The possible explanations for one study were that the pretest scores of self-efficacy were already relatively high and the selection of the instrument had a more general target behavior [25]. The other article studied the comparative effectiveness of Tai Chi and physical therapy, and the control groups were encouraged to perform exercise which may result in nonobvious results [24].

Among the six studies, one pre-post clinical trial [26] showed that no change was found in Tai Chi group after intervention. This may due to the small sample size and lack of follow-up data collection. Additionally, the 6-week Tai Chi intervention may not be long enough to produce significant changes in self-efficacy in patients with KOA and their pre-test self-efficacy to deal with pain was already high [26].

Among the six studies, one RCT even showed that self-efficacy of arthritis decreased at the one-year follow-up compared to eight weeks [27]. Possible reasons for this decrease were that the survey data collected after program and at one-year follow-up were subject to possible recall and self-report bias, and 17% of baseline participants did not return for the eight-week follow-up, which could also bias results.

(2) Two RCTs focused on participants with chronic heart failure, and self-efficacy was measured by different scales. One RCT with a Jadad score of four indicated that exercise self-efficacy significantly improved in the Tai Chi group compared with the control group [17]. The other RCT only had a quality score of two, and it found that self-efficacy of Tai Chi group improved after intervention and no significant difference between the control and Tai Chi groups [31].This may be due to the small sample size; the Tai Chi group and the control group sample size was eight.

(3) Two RCTs studied participants with COPD. One study with a Jadad score of four revealed that both COPD self-efficacy and self-efficacy for managing shortness of breath were significantly improved after six months after intervention in the Tai Chi group [16]. However, another study with a Jadad score of four found that self-efficacy of Tai Chi group improved after intervention and no significant difference between the control and Tai Chi groups [32]. Possible reasons for this include the small sample size and baseline differences between the two groups.

(4) Five studies explored the effects of Tai Chi on self-efficacy in people with other diseases. One RCT with a Jadad score of four focused on sedentary obese women and showed that general self-efficacy was improved in the Tai Chi group and maintained at the 30-week follow-up, but there was no significant differences between two groups [33]. This may be due to the small sample size and the potential lack of statistical power.

Two RCTs with Jadad scores of three [34] and two [35] focused on fibromyalgia patients [34] and Parkinson's patients [35], respectively. Both found significant improvement in the self-efficacy compared with the control groups.

Two other studies examined elderly people with different diseases. One RCT with a Jadad score of three showed that self-efficacy was significantly enhanced in the control and Tai Chi group but no significant differences between two groups [36]. This may be due to relatively small sample size, as this was a pilot study. The other study was a quasi-experimental pre-post study, and it found self-efficacy improved in Tai Chi group but no significant interaction effects between the two groups [37]. The reason for this is that the mean score of self-efficacy at pretest for both group was 80 out of 100, indicating a very high level of self-efficacy.

### 4.3. Effects of Tai Chi on the Self-Efficacy of Different Populations

Under the “population category,” 12 articles analyzed the effects of Tai Chi on college students, elderly people, healthy adult participants, and ethnic Chinese adults with CVD risk factors, indicating inconsistent results.

(1) Five studies focused on the effect of Tai Chi intervention on college students. One RCT [40] with Jadad score of one showed that the self-efficacy significantly improved compared with the control group; one NRS [18] showed significant improvement of Tai Chi groups after intervention but did not mention the comparison result of two groups.

Three studies, including two RCTs with Jadad scores of five [38, 39] and one quasi-experimental study [28], revealed that Tai Chi could not significantly improve self-efficacy compared with control groups. Among the three studies, two RCTs found there were no significant differences between Tai Chi groups and the control groups [38, 39], but one of them showed improvement in the Tai Chi group [39]. Possible reasons could be that all participants enrolled in the two trials were young and apparently healthy college students. Subjects in both groups may have been involved in some regular sporting exercise. It was possible that the 12-week intervention period for Tai Chi exercise was not sufficient to identify significant differences for the young and apparently healthy college student population [38, 39].

Among the three studies, a quasi-experimental study showed no changes between the two groups [41]. A possible explanation was that the Tai Chi group reported higher levels of self-regulatory self-efficacy than the control group prior to intervention [41].

(2) Five studies assessed the effects of Tai Chi on self-efficacy in elderly people. Two RCTs with Jadad scores of one [44] and two [19] studied the population of elderly patients with low activity levels, and they showed significant improvement of Tai Chi groups while compared with the control groups. One RCT [42] with a Jadad score of two found significant improvement in Tai Chi groups in elderly people with disabilities (using wheelchairs) while compared with the control group. One NRS studied elderly people with disabilities, and it showed a significant improvement in Tai Chi groups after intervention [43] but did not mention the comparison result of the two groups.

Among the five studies, one NRS of healthy elderly people [45] found that the Tai Chi group showed no changes after intervention and no significant differences between Tai Chi group and the control group. A possible explanation is that, before the intervention, the Tai Chi group had significantly higher self-efficacy levels than the control group. Additionally, the total sample was small of 33. Furthermore, the participants were already physically active and the measurements of the study may not be appropriate for an active, older population [45].

(3) One RCT with a Jadad score of three [50] and a quasi-experimental study [51] revealed that Tai Chi had positive effects on the self-efficacy of healthy adults and ethnic Chinese adults with CVD risk factors, respectively.

### 4.4. Various Concepts of Self-Efficacy and Measurement Tools

Self-efficacy refers to the beliefs that individuals hold about their capabilities to carry out specific tasks. A belief in one's efficacy leads to successful action, whereas a doubt about one's efficacy causes failure or inaction. Personal efficacy beliefs serve to guide human action in various functional domains [9, 54]. The concept of confidence in one's abilities can be related to a specific domain (specific self-efficacy) or more generally to many stressful situations (general self-efficacy). This systematic review included 27 studies which involve various concepts of self-efficacy and measurement tools. To more clearly describe and compare these studies, we categorized the concept of self-efficacy as general self-efficacy or domain-specific self-efficacy.

#### 4.4.1. General Self-Efficacy and Measurements

General self-efficacy indicates one's optimistic self-beliefs to cope with a variety of difficult demands in life. This systematic review included six studies in which general self-efficacy was measured by five different scales: the General Self-Efficacy (GSE) Scale [40], the GSE Scale developed by Schwazer R in 1992 [49], the GSE Scale developed by Schwazer R in 1995 [50], a Chinese adaptation of the GSE Scale [38], and the Motivation Scale for Health Behaviors [29]. The Motivation Scale for Health Behavior studies the variables of perceived self-efficacy using six items.

#### 4.4.2. Domain-Specific Self-Efficacy and Measurements

In this systematic review, self-efficacy refers to domain-specific efficacy related to fall prevention, pain management, exercise behavior, and other health behaviors.

(1) With regard to fear of falling, falls self-efficacy refers to one's confidence in his/her ability to complete activities without falling. This systematic review included three studies in which falls efficacy was measured by the Falls Self-Efficacy Scale [47], the ABC scale [46], and the MFES [35]. Both the ABC scale and the MFES were extensions and revisions of the Falls Self-Efficacy Scale.

(2) Pain self-efficacy is the belief in one's ability to carry out a range of daily activities despite pain. This systematic review included two studies in which pain self-efficacy was measured by the PSEQ [49] and the CPSS [28]. The PSEQ [49] is a 10-item self-report inventory that measures participants' beliefs about their ability to complete a range of daily activities despite pain. The CPSS [28] is a 22-item questionnaire designed to measure an individual's belief that he/she can cope with the consequences of chronic pain. The CPSS consists of three factors regarding the self-efficacy to cope with pain: self-efficacy for pain management, coping with symptoms, and physical function.

(3) Exercise self-efficacy refers to an individual's perceived confidence in performing certain exercise-related activities. This systematic review included nine studies in which exercise self-efficacy was measured by six scales. The TCSE scale [36] was adopted to assess perceived self-efficacy to overcome barriers to Tai Chi exercise (TCSE barriers) and self-efficacy to perform Tai Chi. The Cardiac Exercise Self-Efficacy Instrument [55] is a 16-item scale that assesses a patient's confidence in performing exercise-related activities on a 5-point scale. The Exercise-related Self-Efficacy Scale [19] measures two aspects of barriers and performance efficacy with regard to exercise. The Self-Regulatory Self-Efficacy Scale [18] was designed to measure self-regulatory self-efficacy and has been correlated with perceived performance and activity-specific self-efficacy. The Self-Efficacy-Barriers to Exercise Scale [48] assessed one's confidence in performing exercise in the face of different barriers. 1-5 Self-Efficacy Scale [15] measured the confidence in one's ability to persist with exercising in different situations.

(4) Arthritis-specific self-efficacy refers to a person's beliefs that one could manage his or her arthritis. In the four studies included in this systematic review, arthritis self-efficacy was measured by the ASES [14], which includes three subscales: arthritis pain self-efficacy, self-efficacy for physical function, and other symptoms. Another study used the Modified Self-Efficacy Scale [37], which included 14 items and was modified by Kim (1994), to measure perceived self-efficacy to cope with arthritis.

(5) This systematic review included two studies in which self-efficacy in COPD was measured by the COPD Self-Efficacy Scale [56], the Chinese version of COPD-CSES [16], and the SEMSOB [16]. The Chinese version of COPD-CSES includes 34 items, evaluating a person's confidence in managing breathing difficulties in different situations. The SEMSOB is a single question on a scale of 1–10, assessing patients' overall confidence in keeping breathing difficulties from interfering with what they want to do.

### 4.5. Comparison of Different Tai Chi Interventions

Tai Chi is not just a form; it is a complete philosophy. Although Tai Chi intervention protocols were all presented in the enrolled 27 studies, the detailed information of Tai Chi (for example, philosophy, stance, and breathing) was not introduced in most of the studies. In the enrolled 27 studies, eleven mentioned breathing very briefly, and there was no further introduction of breathing techniques [15, 17, 23, 24, 26, 31, 32, 34, 36, 43, 44]. Two studies mentioned Tai Chi principles, but further description was lacking [15, 24]. Two studies mentioned stances, one was “stances that require greater than 90° knee flexion” and the other was “the stances are in an upright posture” [11, 27]. Only one study mentioned concentration of the mind and did not have any further description [23]. Philosophy, leg strength, the measurement of deep breathing, flexibility and its measurement, and the measurement of mind were not mentioned in any of the enrolled studies. So, in this systematic review, the styles of Tai Chi, number and frequency of sessions, and the overall length of the intervention were compared based on whether they produced lasting improvements in self-efficacy. Twenty-seven studies were divided into the positive outcome group, the no significant change group, and the negative outcome group. Fifteen studies were included in the positive outcome group, in which self-efficacy significantly improved after Tai Chi intervention. Eleven studies were included in the no change group, in which there was no change in self-efficacy after Tai Chi intervention. Only one study was included in the negative outcome group, in which self-efficacy was decreased significantly.

Tai Chi differentiated into various styles during development and while the Chen style is the oldest, the Yang style is the most popular [2]. In this review, the Yang style was the most commonly adopted (n=9) in the positive outcome group (n=15), although other studies used Sun style (n=1), Chen style (n=1), seated Tai Chi (n=1), and Wheelchair Tai Chi and two studies did not specify the style. Yang style was also the most popular style (n=6) in the no significant change group (n=11), followed by Sun style (n=1), Chen style (n=1), while three studies did not mention the style. Only one study using Sun style was in the negative outcome group. The length of Tai Chi intervention varied from six to 26 weeks in this review. In the positive outcome group (n=15), the most common program length was 12 weeks (n=10), though other studies lasted for 15 weeks (n=1), 24 weeks (n=2), and 26 weeks (n=2). In the no change group (n=11), most interventions were also 12 weeks long (n=6), followed by 6 weeks (n=2), 15 weeks (n=1), 16 weeks (n=1), and 10 weeks (n=1). Only one study that was eight weeks long was reported in the negative outcome group.

The frequency of intervention was one to five sessions weekly. In the positive outcome group (n=15), the most reported frequency of intervention was two sessions weekly (n=10). Other studies held sessions three times weekly (n=3) and four times weekly (n=2). In the no significant change group (n=11), the most common frequency of intervention was two sessions weekly (n=5), with other studies having sessions once weekly (n=3) or five times weekly (n=2). One study had sessions three times weekly for the first two weeks and then weekly for another 10 weeks. The single study in the negative outcome group had two sessions weekly.

The Tai Chi sessions varied in length from 15 to 120 minutes. In the positive outcome group (n=15), most studies (n=9) had 60-minute sessions, with other studies reporting 90 minutes (n=1), 50 minutes (n=1), 40 minutes (n=1), 30 minutes (n=1), or 15 minutes (n=1). One study failed to mention the session length. In the no significant change group (n=11), 60 minutes (n=8) was still the popular session length, though other studies had 50-minute (n=1), 70-minute (n=1), and 120-minute (n=1) sessions. Only one study, with 60-minute sessions, was used in the negative outcome group.

## 5. Conclusions and Limitations

Tai Chi appears to effectively improve self-efficacy among participants with various diseases and across several populations. However, we still could not draw firm conclusions, and this study had some limitations. First, we did not include any unpublished studies and we included different research styles, such as RCT, NRS, quasi-experimental studies, and studies with pre-post design. Second, only seven studies provide follow-up data on participants who continued to practice Tai Chi after the intervention period, and most studies did not mention this; therefore, the long-term effects of Tai Chi on self-efficacy are not clear. Third, the deep mechanism of the effects of Tai Chi on self-efficacy is also unclear. Therefore, we could not judge which types of Tai Chi or which session lengths and intervention durations were most effective. Further studies are needed to optimize effective evidence-based dose-response effects and should include descriptions of intensity, frequency, duration, and adherence of the Tai Chi exercise. In addition, considering the principle of philosophy as well as stances, deep breathing, flexibility, leg strength, and mind is the essence of Tai Chi, which could be explored deeply and in detail in future researches; and future studies can explore the exacted principles and techniques of the Tai Chi protocol.

In addition, the studies reviewed here involved a wide variety of Tai Chi styles, frequency, duration, and follow-up, and many different scales were used to measure different concepts of self-efficacy. These studies also adopted different research strategies, and their statistical analysis methods and data were often presented in the raw form; it was therefore not possible to perform a meta-analysis.

## Figures and Tables

**Table 1:** Basic characteristics of the included studies.

Reference (year)	Country	Subjects, N	Age (Tai Chi/ Controls)	Study Design	Intervention Frequency, style		Intervention Duration	Outcome Measured	Outcomes	quality Score (randomization/ blinding/ dropouts)
					Tai Chi, N	Control(s), N				
**1 Disease category**										
**1.1 Arthritis**										
Chenchen Wang, et al (2009) [15]	the United States	KOA, 40	65±7.8	RCT	60 min, 2x/wk, 10- form classic Yang style, 20	education and stretching program, 20	12 weeks	1-5 Self Efficacy Scale	Self-efficacy significantly improved in Tai Chi group vs. control group	2/1/1

Catherine A. Hartman, et al (2000) [23]	the United States	osteoarthritis, 33	68	RCT	60 min, 2x/wk, 9-form Yang style, 18	usual activities, 15	12 weeks	Arthritis Self Efficacy Scale	Self-efficacy for arthritis symptoms and total arthritis significantly improved in Tai Chi group vs. control group	2/1/ND

Chenchen Wang, et al (2016) [24]	the United States	KOA, 204	60	RCT	60 min, 2x/wk, Yang style, 106	physical therapy, 98	12 weeks	Arthritis Self Efficacy Scale	Self-efficacy improved in Tai Chi group, but no significant difference in two groups	2/1/1

Rhayun Song, et al (2007) [25]	Korea	osteoarthritis, 72	63	RCT	first two weeks, 60 min, 3x/wk; another 10 weeks, 1x/wk; 12-form Sun style, 38	the control group, 34	12 weeks	The Motivation Scale for Health Behaviors	Self-efficacy improved after Tai Chi intervention, but no significant difference between two groups	2/ND/1

Chwan Li Shen, et al (2008) [26]	the United States	KOA, 40	64.4 ± 8.3	Pre-Post study	60 min, 2x/wk, 24-form simplified Yang style	/	6 weeks	Chronic Pain Self Efficacy Scale (CPSS)	No changes were found in self-efficacy of pain management, physical function and other symptoms	/

Leigh F. Callahan, et al (2016) [27]	the United States	arthritis, 343	66	RCT	60 min, 2x/wk, Sun Style, 181	wait-list control, 162	8 weeks	Arthritis Self Efficacy Scale	Arthritis self-efficacy of pain and other symptoms had no difference at 8 weeks but decreased at one-year follow up	2/ND/1

**1.2 Chronic Heart Failure**										
Gloria Y Yeh, et al (2011) [17]	the United States	chronic systolic heart failure, 100	67±11	RCT	60 min, 2x/wk, adapted from Master Cheng Man-Ch'ing's Yang style short form, 50	education attention control, 50	12 weeks	the Cardiac Exercise Self-Efficacy Instrument	Self-efficacy significantly improved in Tai Chi group vs. control group	2/1/1
Gloria Y Yeh, et al (2013) [31]	the United States	heart failure with preserved ejection fraction, 16	66±12	RCT	60 min, 2x/wk, adapted from Master Cheng Man-Ch'ing's Yang-style short form, 8	aerobic exercise control, 8	12 weeks	The Self-Efficacy- Barriers to Exercise Scale	Self-efficacy improved in Tai Chi group but no significant difference between two groups	2/ND/ND

**1.3 COPD**										
Lorna Ng, et al (2014) [16]	Hong Kong	COPD, 192	74.16± 6.46 74.13± 6.81	RCT	pulmonary rehabilitation program and 15 min, 2x/wk, 5-form Sun Style, 94	pulmonary rehabilitation program, 98	12 weeks	COPD Self Efficacy Scale (COPD-CSES); Self Efficacy for Managing Shortness of Breath (SEMSOB)	COPD-CSES and SEMSOB significantly improved in Tai Chi group at 6-month but no significant difference between two groups	2/1/1
Gloria Y Yeh, et al (2010) [32]	the United States	COPD, 10	65±6 66±6	RCT	Tai Chi plus usual care 60 min, 2x/wk, Master Cheng Man-Ch'ing's Yang style short form, 5	usual care, 5	12 weeks	COPD-CSES	Self-efficacy improved in Tai Chi group but no significant difference of two groups	2/1/1

**1.4 other diseases**										
Arnaud Dechamps, et al (2009) [33]	France	sedentary obese women, 21	44.4±11.9	RCT	Diet plus TC, 2-hour weekly group session Yang style, 11	Diet plus conventional structured exercise program, 10	10 weeks	the General Self Efficacy (GSE) Scale	General self-efficacy improved in Tai Chi group but no significant difference between two groups	2/1/1
Kim D. Jones, et al (2012) [34]	the United States	fibromyalgia, 98	54	RCT	90 min, 2x/wk, 8-form Yang style, 51	education control, 47	12 weeks	Arthritis Self Efficacy Scale	Self-efficacy for pain control, function and other symptoms significantly improved vs. control group	2/ND/1
Bo Li, et al (2017) [35]	China	Parkinson, 60	ND	RCT	60 min, 4x/wk, Simplified 24-Form, 30	usual care, 30	12 weeks	the Modified Falls Efficacy Scale (MFES)	Fall self-efficacy in Tai Chi group significantly improved vs. control group	1/ND/1
Arnaud Dechamps, et al (2009) [36]	France	elderly with different diseases, 52	80.7±8.9	RCT	30 min, 4x/wk, Yang style, 26	Cognition action exercise program, 26	24 weeks	Falls Efficacy Scale (FES); TC exercise self efficacy (TCSE) scale	Self-efficacy significantly improved in Tai Chi group but no significant difference between two groups	1/1/1
Lee, Eunhee (2010) [37]	Korea	Korean American older women with different diseases, 41	65.8±6.6 63.1±7.9	pre-post study	60 min, weekly, plus Health education, 20	Health education, 21	16 weeks	Self Efficacy Scale	Self-efficacy improved in Tai Chi group but no significant difference between two groups	/

**2 Population category**										
**2.1 College Students**										
Guohua Zheng, et al (2015) [38]	China	college students, 198	20.6±1.1	RCT	60 min, 5x/wk, 24-form simplified Tai Chi, 95	usual physical activities , 103	12 weeks	Chinese adaptation of the General Self efficacy Scale	No significant changes were found after Tai Chi intervention and the comparison of two groups	2/2/1
Ting Rao,(2014) [39]	China	college students, 206	16-25	RCT	60 min, 5x/wk, 24-form simplified Tai Chi, 103	Blank control, 103	12 weeks	General Self Efficacy Scale	Self-efficacy improved in Tai Chi group after 12 weeks but no significant difference in two groups	2/2/1
Wei Sun,(2016) [40]	China	college students, 60	ND	RCT	2x/wk, 30	other activities, 30	12 weeks	General Self Efficacy Scale	Self-efficacy significantly improved after 12 weeks vs. control group	1/ND/ND
Karen Caldwell, et al (2011) [41]	the United States	college students, 208	18-48	quasi- experimental study	50 min, 2x/wk, Chen-style Tai Chi classes, 76	the special recreation, 132	15 weeks	The Self- regulatory Self- Efficacy Scale	Self-efficacy showed no significant difference in two groups	/
Karen Caldwell, et al (2011) [18]	the United States	college students, 127	18-32	NRS	50 min, 2x/wk, Chen-style, 35	Pilates mat classes, two recreation classes, 92	15 weeks	The Self- regulatory Self- Efficacy Scale	Self-efficacy significantly improved in Tai Chi group but no comparisons between two groups	/

**2.2 Elderly People**										
Chen-Yuan Hsu, et al (2016) [42]	Tai Wan	older Taiwanese people using wheelchairs, 60	80.73±9.68	RCT	40 min, 3x/wk, seated simplified Tai Chi exercise program (STEP), 30	usual exercise and entertainment activities group, 30	26 weeks	Self- Efficacy for Exercise Scale	Self-efficacy significantly improved vs. control group	2/ND/ND
Yong Tai Wang, et al (2016) [43]	the United States	elderly with disability, 28	87.23±6.71 89.73±6.31	NRS	60 min, 2x/wk, Wheelchair Tai Chi (WTC), 13	control group, 15	12 weeks	Pain Self-Efficacy Questionnaire (PSEQ)	Self-efficacy significantly improved in Tai Chi group but no comparisons between two groups	/
Fuzhong Li, et al (2005) [44]	the United States	inactive older adults, 256	77.48±4.95	RCT	60 min, 3x/wk, 24-Form Yang style, 125	Stretching control exercise condition, 131	26 weeks	Activities- Specific Balance Confidence (ABC) Scale	Tai Chi significantly improved falls self efficacy over 26 weeks follow-up vs. the control	1/ND/ND
Fuzhong Li, et al (2001) [19]	the United States	inactive older adults, 94	72.8±5.1	RCT	60 min, 2x/wk, 24-Form Yang style, 49	waiting list control group, 45	24 weeks	Exercise- related self-efficacy Scale	Tai Chi improved barriers efficacy and performance efficacy significantly vs. control group	1/ND/1
Busing, J Kyle (2005) [45]	the United States	healthy elderly adults, 33	69±5.7	NRS	70 min, weekly, Yang style 24-Form Simplified Tai Chi, 15	the exercise group, 18	6 weeks	Falls Self- Efficacy Scale	No significant changes were found after Tai Chi intervention and the comparison of two groups	/

**2.3 Other population**										
Marko Nedel jkovic, et al (2013) [50]	Switzerland	healthy adults, 70	35.86±8.64 35.13±6.53	RCT	60 min, 2x/wk, Man-Ch'ing's Yang-Style short form, 35	waiting list control group, 35	12 weeks	Generalized Self- Efficacy Scale	Self-efficacy improved significantly in Tai Chi group vs. control group	2/ND/1
Ruth E. Taylor Piliae, et al (2006) [51]	the United States	ethnic Chinese adults with cardiovascular disease risk factor, 39	66±8.3	quasi- experimental study	60 min, 3x/wk, Yang Style 24-posture short-form	/	12 weeks	TCSE Scale	Self-efficacy to overcome barriers and confidence to perform Tai Chi significantly improved	/

N=number of subjects; wk(s), week(s); min(s), minute(s); ND=no data; vs.=versus.

**Table 2:** Studies with positive results.

Reference	study design		Tai Chi group		control group		P value		
			baseline	after intervention	baseline	after intervention			
Catherine A. Hartman, et al [23]	RCT	self-efficacy for arthritis symptoms	70.6 (13.5)	81.6 (9.5)	79.0 (12.1)	80.3 (11.4)	P=0.012	M (SD)	between groups
		total arthritis self-efficacy	220.0 (39.3)	242.5 (28.5)	231.5 (27.6)	231.4 (32.5)	P=0.043		
Gloria Y Yeh, et al [17]	RCT	exercise self-efficacy	3.6 (2.7,3.8)	3.7 (3.5,4.1)	3.7 (3.1,4.3)	3.4 (3.0,4.0)	P<0.001	Median (Q1,Q3)	between groups
Chen-Yuan Hsu, et al [42]	RCT	exercise self-efficacy	33.26 (32.13)	35.66 (36.83)	27.16 (29.06)	15.30 (26.43)	P=0.01	M (SD)	between groups
Fuzhong Li, et al [44]	RCT	fall self-efficacy	7.59 (1.09)	8.65 (1.20)	7.65 (1.21)	7.82 (1.20)	P<0.001	M (SD)	between groups
Fuzhong Li, et al [19]	RCT	barrier efficacy	38.575 (10.713)	42.888 (7.107)	39.531 (8.744)	31.844 (12.295)	P<0.05	M (SD)	between groups
		performance efficacy	23.225 (6.867)	26.825 (4.574)	23.000 (6.849)	21.344 (8.560)	P<0.05		
Wei Sun, [40]	RCT	general self- efficacy	30.1433	32.3561	32.0767	32.1032	P<0.01	M	between groups
Bo Li, et al [35]	RCT	fall self-efficacy	4.81 (1.39)	7.73 (1.36)	4.74 (1.25)	5.64 (1.32)	P<0.001	M (SD)	between groups
Lorna Ng, et al [16]	RCT	CSES	0.638 (0.152)	0.685 (0.137)	0.684 (0.165)	0.733 (0.143)	P<0.001	M (SD)	within groups
		SEMSOB	6.53 (1.98)	7.03 (1.75)	6.86 (2.34)	7.13 (1.98)	P<0.001		
Karen Caldwell, et al [18]	NRS	general self- efficacy	57.6	63.2	/	/	P=0.0005	M	within groups
Yong Tai Wang, et al [43]	NRS	PSEQ	54.58 (6.72)	56.25 (5.49)	43.67 (14.97)	43.13 (16.05)	P<0.05	M (SD)	within groups
Arnaud Dechamps, et al [36]	RCT	fall self-efficacy	55.1 (23.44)	16.8 (7)	48.6 (26.3)	26.5 (23.6)	P<0.001	M (SD)	within groups
		exercise self-efficacy	40.3 (21.4)	56.3 (32.4)	35.3 (21.3)	63.9 (33.7)	P<0.003	M (SD)	within groups
Chenchen Wang, et al [15]	RCT	arthritis self-efficacy -week 12	0.60 (0.12, 1.08)		−0.11 (−0.59, 0.37)		P=0.04	M (95%CI) change from baseline	between groups
		arthritis self-efficacy -week 24	0.68 (0.20, 1.16)		−0.17 (−0.65, 0.31)		P=0.02		
		arthritis self-efficacy -week 48	0.72 (0.24, 1.20)		−0.24 (−0.72, 0.24)		P=0.007		
Marko Nedel jkovic, et al [50]	RCT	general self- efficacy -week 12	1.89 (2.88)		−0.10 (2.52)		P=0.006	M (SD) change from baseline	between groups
		general self- efficacy -week 20	2.46 (2.30)		1.06 (2.58)		P=0.033		
Kim D. Jones, et al [34]	RCT	self-efficacy-pain	9.2 (2.1, 18.3)		−1.5 (−0.7, −0.2)		P=0.00001	M (95%CI) change from baseline	between groups
		self-efficacy-function	7.9 (0.9, 14.1)		−0.3 (−0.5, −0.1)		P=0.00007		
		self-efficacy- other symptoms	12.5 (3.8, 21.1)		−0.8 (−0.9, −0.6)		P=0.00001		
Ruth E. Taylor Piliae, et al [51]	quasi-experimental study	TCSE barriers	25.0 (37.1)				P<0.01	M (SD) change from baseline	within groups
		TCSE performance	18.8 (26.3)				P<0.01		

Q= quartile; M=mean; SD=standard deviation; 95%CI=95% confidence interval.

**Table 3:** Studies of no significant changes and negative group.

Reference	study design		Tai Chi group		control group		P value		
			baseline	after intervention	baseline	after intervention			
Gloria Y Yeh, et al [31]	RCT	exercise self-efficacy	59.6 (30)	66.6 (29)	50.3 (24)	53 (28)	P=0.18	M (SD)	between groups
Gloria Y Yeh, et al [32]	RCT	COPD self-efficacy	105 (87-149)	135 (102-137)	135 (129-143)	137 (111-144)	P=0.20	Median (range)	between groups
Rhayun Song, et al [25]	RCT	self-efficacy	17.47 (4.03)	18.12 (3.67)	17.27 (2.45)	17.20 (3.18)	P=0.55	M (SD)	between groups
Arnaud Dechamps, et al [33]	RCT	general self- efficacy -week 10	27.6 (6.6)	35.4 (3.5)	29.3 (5.7)	35.2 (6.4)	/	M (SD)	between groups
		-week 30	27.6 (6.6)	35 (2.4)	29.3 (5.7)	31.3 (4)	/	M (SD)	
Lee, Eunhee [37]	pre-post study	self- efficacy-week 8	80.8 (14.2)	81.2 (13.1)	79.5 (20.6)	79.9 (14.9)	P=0.487	M (SD)	between groups
		self-efficacy-week 16	80.8 (14.2)	85.5 (13.6)	79.5 (20.6)	81.9 (16.5)			
Ting Rao, [39]	RCT	general self- efficacy	2.55 (0.43)	2.59 (0.46)	2.47 (0.39)	2.54 (0.46)	P>0.05	M (SD)	between groups
Guohua Zheng, etal [38]	RCT	self-efficacy	2.56 (0.43)	2.59 (0.44)	2.47 (0.39)	2.54 (0.45)	P>0.05	M (SD)	between groups
Chenchen Wang, et al [24]	RCT	arthritis self- efficacy-week 12	1.20 (1.09-1.32)		1.14 (1.03-1.26)		P=0.73	M (95%CI) change from baseline	between groups
		-week 24	1.12 (1.02-1.24)		1.16 (1.04-1.29)				
		-week 52	1.14 (1.04-1.25)		1.13 (1.03-1.24)				
Chwan Li Shen, et al [26]	pre-post study	self-efficacy-pain	60.3 (37.4)		67.3 (22.5)		P=0.291	M (SD)	within groups
		self-efficacy-function	67.3 (40.1)		76.1 (21.7)		P=0.619		
		self-efficacy-other symptoms	63.1 (37.3)		70.5 (20.3)		P=0.714		
Busing,J Kyle [45]	NRS	fall self-efficacy	12.45 (3.88)	/	12.60 (4.42)	/	P>0.98	M (SD)	within groups
Karen Caldwell, et al [41]	quasi- experiment	general self-efficacy	20.76 (3.39)	/	19.56 (2.96)	/	/	M (SD)	between groups
Leigh F. Callahan, et al (2016) [25]	RCT	arthritis self-efficacy-pain -week 8	7.35 (1.55)	7.48 (2.08)	6.99 (1.90)	7.05 (1.92)	/	M(SD)	between groups
		arthritis self-efficacy-other symptoms -week 8	7.42 (1.72)	7.60 (2.30)	7.23 (1.96)	7.09 (1.92)	/	M(SD)	between groups
		arthritis self-efficacy-pain -week 8 to 1 Year	-0.38 (-0.61, -0.14)	/	P<0.01	Mean Change (95% CI)	within groups		
		arthritis self-efficacy-other symptoms -week 8 to 1 Year	-0.39 (-0.67, -0.12)	/	P<0.01	Mean Change (95% CI)	within groups		

M=mean; SD=standard deviation; 95%CI=95% confidence interval.
